# Equine Bone Marrow–Derived MSCs and Their EVs Exhibit Different Immunomodulatory Effects on Cartilage Explants in an *In Vitro* Osteoarthritis Model

**DOI:** 10.1177/19476035251378693

**Published:** 2025-09-25

**Authors:** Angela M. Gaesser, Justine M. Cianci, Kayla Even, Renata L. Linardi, Gordon Ruthel, Dhvani Barot, Hoda Elkhenany, Julie B. Engiles, Kyla F. Ortved

**Affiliations:** 1 Department of Clinical Studies, New Bolton Center, University of Pennsylvania, Kennett Square, PA, USA; 2 Department of Clinical Sciences and Advanced Medicine, New Bolton Center, University of Pennsylvania, Kennett Square, PA, USA; 3 Department of Pathobiology, University of Pennsylvania, Philadelphia, PA, USA

**Keywords:** osteoarthritis, exosome, microvesicle, extracellular vesicle, mesenchymal stem cell, immunomodulation

## Abstract

**Objective:**

To compare the effects of equine MSCs and their extracellular vesicles (EV) on stimulated cartilage explants and assess how serum type influences EV production and cartilage inflammation.

**Methods:**

EVs were isolated from bone marrow–derived MSCs cultured in equine serum (ES) or fetal bovine serum (FBS) media and concentrated via ultracentrifugation. Cartilage explants were stimulated with IL-1β and TNF-α and treated with MSCs, EVs, or left untreated. Cartilage explants were analyzed for cytokine concentration and examined for gene expression, glycosaminoglycan depletion, and histology.

**Results:**

EVs produced by MSCs cultured in ES or FBS had similar characteristics. Cartilage explants treated with MSCs in ES media had decreased concentrations of IL-1β and increased concentrations of IL-6 in the supernatant compared to cartilage explants alone. Treatment with EVs did not significantly alter supernatant mediators. Cartilage explants cultured in ES had higher levels of IL-1β, IL-6, and TNF-α, while cartilage explants cultured in FBS had higher levels of PGE2. Treatment of stimulated cartilage explants with either MSCs or EVs did not alter gene expression or support extracellular matrix (ECM) degradation.

**Conclusion:**

Equine MSCs appear to have enhanced immunomodulatory properties compared to EVs when used to treat stimulated cartilage explants. While some beneficial alterations in culture supernatants were detected, ECM degradation was not affected by treatment.

## Background

Osteoarthritis (OA) is the most common joint disease worldwide, affecting an estimated 10% of men and 18% of women over 60 years of age, contributing significantly to long-term disability and causing a notable healthcare and socioeconomic burden.^1,2^ Inflammation is widely accepted as a contributing factor in the initiation and progression of OA. Synoviocytes in the synovial membrane and chondrocytes within the articular cartilage play a pivotal role in joint inflammation, producing several inflammatory cytokines, including interleukin (IL)-1β, IL-6, and tumor necrosis factor (TNF)-α, and matrix-degrading enzymes including the metalloproteinases (MMP) and a disintegrin and metalloproteinase with thrombospondin-like motifs (ADAMTS), when activated.^3,4^ The inflammatory cascade leads to breakdown of the cartilage extracellular matrix (ECM), further driving inflammation and joint degradation, which releases glycosaminoglycans (GAGs).^5^

Current OA treatment mainly includes intra-articular corticosteroid administration, intra-articular hyaluronic acid, and non-steroidal anti-inflammatory medication, among other therapeutic and lifestyle changes.^6^ However, these treatments are non-curative and do not restore cartilage to its previous condition, rather aiming for pain reduction and symptom control. Alternative therapies focusing on immunomodulation, inflammation control and regeneration have garnered significant interest in both human and veterinary medicine. Regenerative medicine seeks to restore the normal function of diseased or damaged cells, tissues, and organs through various approaches, including cell-based therapies, which are designed to stimulate and coordinate the processes involved in biological repair.

Bone marrow-derived mesenchymal stem cells (BM-MSC) have been widely investigated as a therapy for multiple orthopedic diseases, including tendon/ligament injuries, OA, and cartilage lesions in various species.^7-10^ MSCs can exert their immunomodulatory effect via paracrine signaling through the secretion of anti-inflammatory and regenerative factors largely packaged in extracellular vesicles (EV).^11-13^ EVs can transport packaged nucleic acids, lipids, and proteins to cells, therefore, in theory, recreating the therapeutic effects of the MSCs without the administration of cells. BM-MSC derived EVs and BM-MSCs have been reported to exert similar chondroprotective and anti-inflammatory effects *in vitro* and protected mice from developing OA *in vivo*, suggesting that the EVs reproduced the same therapeutic effect of the parent BM-MSCs.^13^ Other animal models have been used extensively in the study of OA.^14^ However, horses provide a relevant large animal model of OA due to the common occurrence of naturally occurring OA, as well as significant similarities in^15,16^ cartilage thickness, biochemical composition and biomechanical loading when compared to human joints,^17^ and consistently predictable *in vivo* and *in vitro* models^18,19^ to study pathobiological events.

The traditional method of MSC culture expansion uses media supplemented with fetal bovine serum (FBS), which supplies essential growth factors and nutrients. However, the potential immunogenicity of FBS-supplemented cells raises concern, as xenogeneic antigens in the FBS can activate the immune system and lead to cell rejection.^20,21^ Immunosuppression or tolerizing regimens have been used in mouse models to circumvent immunogenicity of stem cell therapy as well as in human stem cell transplant recipients.^21^ In addition, FBS proteins can be internalized or bound to the extracellular matrix of MSCs during culture.^22^ Despite efforts to remove FBS proteins, some residual levels remain detectable.^22,23^ Researchers seeking alternatives to FBS found that serum-free media altered the immunomodulatory properties of the cells by decreasing secretion of prostaglandin E2 (PGE2) and increasing secretion of IL-10.^24^ A similar study culturing human BM-MSCs in different types of commercially available reduced-serum media found variability in vascular endothelial growth factor (VEGF) production, differentiation potential, and immunosuppressive properties.^25,26^ A recent study found that equine BM-MSCs cultured in xenogen-free media supplemented with equine serum were more effective at suppressing T-cell proliferation compared to cells cultured in FBS.^27^

To our knowledge, no studies have compared the effects of xenogen-free media on the chondroprotective effects of equine BM-MSC EVs. The objectives of this study were to compare the effects of equine BM-MSC EVs and BM-MSCs, as well as serum supplement type, on stimulated cartilage explants. We hypothesized that BM-MSC EVs, would have chondroprotective effects on induced stimulated cartilage *in vitro* and that these effects would be superior to BM-MSCs. In addition, we hypothesized that BM-MSC EVs harvested from cells expanded in ES would be more chondroprotective than those expanded in FBS.

## Materials and Methods

### Animals

**Figure 1.**
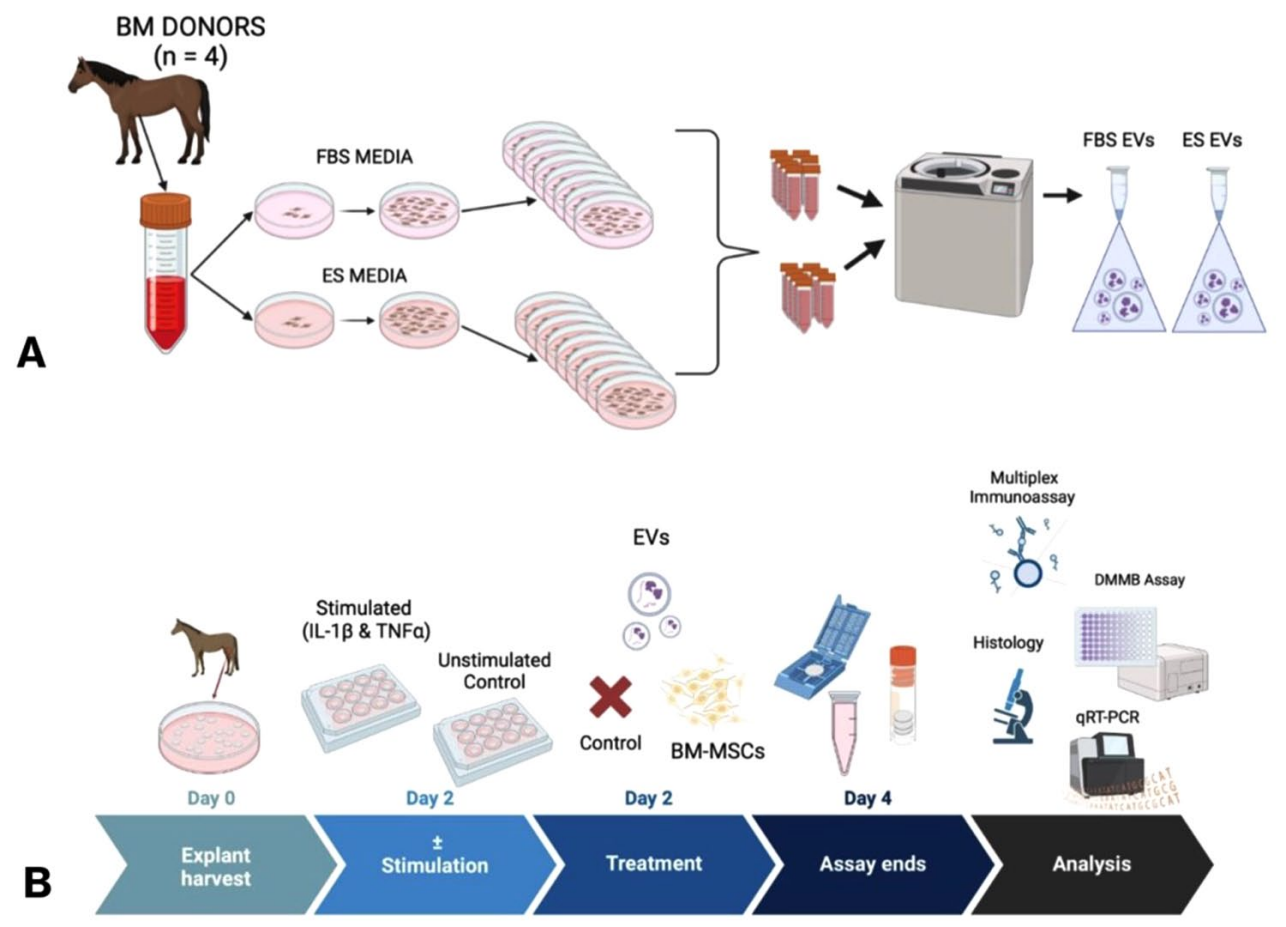
Overview of the study design. (**A**) Bone marrow was collected (*n* = 4) and mesenchymal stem cells (MSC) were culture-expanded in either fetal bovine serum (FBS)-supplemented media or equine serum (ES)-supplemented media. Extracellular vesicles were then isolated via stepwise centrifugation. (**B**) Cartilage explants were collected from the lateral trochlear ridge of healthy horses (*n* = 4), cultured in ES or FBS-supplemented media, and stimulated with IL-1β and TNF-α or left unstimulated for 48 hours. Cartilage explants were then treated with MSCs, EVs, or left untreated for an additional 48 hours. Following the culture period, supernatants and cartilage explants were collected for downstream analyses including supernatant protein quantification, glycosaminoglycan quantification, histology, and cartilage explant gene expression.

Bone marrow was collected from the sternum of 4 healthy, adult horses (median age = 7.5 years, 3-12 years). Horses included 3 geldings and 1 mare. Four different healthy, adult horses (median age=6.5, 4-10 years) served as cartilage donors. Horses included 3 geldings and 1 mare. The study was performed per Institutional and NIH guidelines for the Care and Use of Laboratory Animals and approved by the Institutional Animal Care and Use Committee (IACUC) at the University of Pennsylvania. An overview of the study design is shown in **Figure 1**.

### Bone Marrow Collection and BM-MSC Culture

Bone marrow was collected aseptically from the sternebrae of standing, sedated horses. Briefly, 30 mL of bone marrow was aspirated using an 11-gauge Jamshidi™ Bone Marrow Biopsy Needle (VWR Scientific, Bridgeport, NJ, USA) and 60-mL syringe containing 10,000 IU of heparin. Bone marrow was washed with complete media and centrifuged at 1,500 rpm for 10 minutes at 4°C. The supernatant was removed and the remaining 20 mL containing bone marrow cells were seeded into a T-175 cell culture flask containing culture media. The media consisted of Dulbecco’s Modified Eagle Medium (DMEM) with 1 g/L of d-glucose, 2 mM l-glutamine, and 1 mM sodium pyruvate (ThermoFisher Scientific, Hampton, NH), penicillin (100 U/mL)-streptomycin (100 µg/mL) solution (Invitrogen, Carlsbad, CA), HEPES (ThermoFisher Scientific, Hampton, NH) and either: (1) 10% Fetal Bovine Serum (FBS) (VWR Life Science Seradigm, VWR, Radnor, PA) and human basic fibroblast growth factor (bFGF) (1 ng/mL) (Invitrogen, Carlsbad, CA) or (2) 10% equine serum (ES; New Zealand origin, ThermoFisher Scientific, Hampton, NH) and equine bFGF (1 ng/mL). Media was changed every 48 hours. Cultured cells were passaged when they reached ~80% confluency using Trypsin-EDTA Cell Dissociation Reagent (ThermoFisher Scientific, Waltham, MA, USA). Cell number and viability were determined using the Cellometer Auto 2000 Cell Viability Counter (Nexcelom Bioscience, Lawrence, MA) and ViaStain™ AOPI staining solution (Nexcelom Bioscience LLC, Lawrence, MA).

### EV Collection and Isolation

Passage 3 (P3) cells were used for EV isolation. At 80% cell confluency, the culture media was replaced with EV depleted media as previously described.^28^ Briefly, FBS and ES were filtered through a centrifugal filter unit with a 100 kDa molecular weight cut-off filter (Amicon® Ultra – 15, Ultracel® – 100K, Millipore Sigma) by centrifugation at 3,000 xg for 55 minutes. The EV depleted FBS and ES were then added to the cell culture media and cells were cultured for an additional 48 hours. Supernatants were then collected into 50 mL centrifuge tubes and EVs were collected using differential ultracentrifugation. Briefly, the tubes were centrifuged at 100,000 xg for 120 minutes at 4°C and the supernatant discarded. The remaining pellets were then resuspended in PBS, transferred to a new microcentrifuge tube, and centrifuged at 100,000 xg for 120 minutes at 4°C. All pellets were resuspended in a final 100 mL filtered PBS and stored at −80°C until further use.

### EV Characterization

Nanoparticle tracking analysis (NTA) was performed to measure hydrodynamic particle size and concentration (ZetaView® PMX220 Twin, Particle Metrix) as previously described.^29^ For each sample, 11 positions were scanned, and 30 frames captured per position. The following settings were used: camera sensitivity: 80; shutter: 100; cell temperature: 25°C. The videos were analyzed by ZetaView Software version 8.05.12 with a maximum particle size of 1,000 nm, a minimum particle size of 10 nm, and a minimum particle brightness of 30. The laser wavelength was set to 488 nm. Particle size distribution data was sorted into 3 categories based on size ranges of EV subtypes: exosomes (0 to 99 nm), microvesicles (100 to 1,000 nm), and apoptotic bodies (1,001 to 6,000 nm). The proportion of each subtype was then calculated by dividing the number of EVs in each category by the total number of EVs counted.

Transmission electron microscopy (TEM) was used to evaluate EV morphology as previously described.^29^ A 5 µl volume of EV sample was applied to a thin carbon grid that was glow discharged for 2 minutes using a PELCO easiGlow™ instrument (Ted Pella, Inc., Redding, CA). Next, 5 µL of freshly made 2% uranyl acetate stain solution was applied to the sample holding grid and incubated for 2 minutes. Excess sample and stain were blotted away with filter paper leaving a thin layer of stained particles on the grid. The staining process was repeated, and the grid was left to dry until imaged. TEM micrographs were collected using a Tecnai T12 TEM microscope at 100 keV. The images were recorded on a Gatan Oneview 4K x 4K camera. Each image was collected by exposing the sample for 4 seconds and a total of 100 dose-fractionated images were collected and averaged into a single micrograph. The data was collected at −1.5 to 2 µm under focus at 30K to 40K magnification.

Western blot (WB) was used to detect specific EV markers as previously described.^25^ Extracellular vesicle samples (*n* = 4; 6 x 10^7^ particles) containing 2x Laemmli loading buffer (Bio-Rad Cat.#161-0737) were loaded into a 4% to 12% pre-casted gel (NuPAGE™, Invitrogen, total volume of 20-30 mL per well) in a non-reducing condition. Precision Plus Protein™ Kaleidoscope™ Prestained Protein Standards (Bio-rad, #1610375, 5 mL/well) was used as molecular weight markers. Electrophoresis was initially run at 80 V for 20 minutes and then at 150 V for 60 minutes. The proteins were transferred from the gel to a polyvinylidene difluoride (PVDF) membrane (Immobilon-P, Millipore, #IPVH00010) at 70 V for 2 hours. After transfer, the membrane was briefly rinsed in tris-buffered saline (TBS) solution and blocked with 5% dry skim milk for 2 hours at room temperature (RT) before incubation with a primary antibody overnight at 4°C. Primary antibodies included anti-CD9 (Biolegend, #312102), anti-CD81 (Santa Cruz Biotechnology, #sc-166029 HRP), and anti-TSG101 (Millipore Sigma, #AV38773). Anti-calnexin (Millipore Sigma, #AB2301) was used as a negative marker. After incubation, the membrane was washed with TBS+Tween 20 (TTBS) at RT (3 times for 5 minutes each) and then incubated with horseradish peroxidase (HRP) secondary antibody for 1 hour at RT. Secondary antibodies included goat anti-mouse for CD9 and CD81 (Biolegend, #405306) and donkey anti-rabbit for TSG101 and Calnexin (Biolegend, #406401). The membrane was washed with TTBS for 2 hours, changing the wash every 15 to 20 minutes, and then incubated with Immobilon Forte Western HRP Substrate (Millipore, #WBLUF0100) for 2 minutes at RT for chemiluminescence detection. Detection was performed using the Amersham ImageQuant™ 800 imaging system (Cytiva, Marlborough, MA) after 30 to 60 seconds of exposure time.

### Cartilage Collection and Cartilage Explant Cultures

Cartilage explants were harvested under sterile conditions using a 6 mm biopsy punch from the femoral lateral trochlear ridges of 4 horses euthanized for reasons unrelated to the study. Explants were cultured in cartilage media containing Ham’s F12 medium supplemented with 50 µg/mL ascorbic acid, 30 µg/mL a-ketoglutarate, 300 µg/mL L-glutamine, 100 U/mL penicillin/ streptomycin, 25 mM HEPES, and either 10% FBS or 10% ES for 48 hours. Passage 3 BM-MSCs were plated in a 12-well transwell plate at a seeding density of 10,000 cells/cm^2^ and cultured for 24 hours prior to addition of cartilage explants. After 24 hours, cell culture media was replaced with cartilage media containing either 10% FBS or 10% ES and cartilage explants were transferred to the top of the transwell inserts (4 explants/insert).^30^ EVs were added to the appropriate wells at 1 x 10^9^ EVs per well. The following treatment groups were examined in duplicate: (1) MSCs co-cultured with cartilage explants in FBS media; (2) MSCs co-cultured with cartilage explants in ES media; (3) EVs from MSCs co-cultured with cartilage explants in FBS media; (4) EVs from MSCs co-cultured with cartilage explants in ES media; (5) Cartilage explant only in FBS media; and (6) Cartilage explant only in ES media. All treatment groups were either stimulated with IL-1β (10 ng/mL) and TNF-α (1 ng/mL) or left unstimulated as previously described. After a 48-hour culture, supernatants and cartilage explants were harvested for downstream analyses. Supernatants were aliquoted into microcentrifuge tubes and stored at −20°C.

### Supernatant Analysis

The concentration of select immunomodulatory cytokines including (IL-1β, IL-6, IL-10, and TNF-α) using an equine specific fluorescent bead-based multiplex immunoassay (Milliplex^®^, Millipore, Burlington, MA) on the Luminex^®^ 200 instrument (Luminex, Austin, TX) in accordance with the manufacturer’s instructions and as previously described.^26^ Briefly, 25 µL of standard, control, or sample were added to a well followed by 25 µL of antibody-immobilized beads. Plates were incubated overnight at 4°C on a microplate shaker, then washed 3 times before adding 25 µL of detection antibody to each well. Plates were then incubated at RT on a microplate shaker for 1 hour before adding 25 µL of streptavidin-phycoerythrin to each well and incubating at RT on a microplate shaker for 30 minutes. The plate was washed 3 times and then 150 µL of drive fluid was added to each well prior to analysis. Parameters for analysis on the Luminex^®^ 200 instrument with xPONENT^®^ software were set at 50 events per bead and a sample size of 100 µL.

The concentration of PGE2 was quantified using a competitive enzyme-linked immunoassay (#KGE004B, R&D Systems, Minneapolis, MN) according to manufacturer’s instructions. Briefly, 150 µL of standard, control or sample was added to the appropriate well followed by addition of 50 µL of primary antibody solution. The plate was incubated on a microplate shaker at RT for 1 hour and then 50 µL of conjugate was added to each well. The plate was incubated again on a microplate shaker at RT for 2 hours and then washed 4 times. Next, 200 µL of substrate solution was added to each well and the plate was incubated at RT for 30 minutes. Stop solution (100 µL) was added and the optical density measured within 30 minutes using a microplate reader at 450 nm with a wavelength correction set at 570 nm.

### Glycosaminoglycan Quantification

Glycosaminoglycan (GAG) content in the culture supernatants and cartilage explants using the dimethylenemethylene blue (DMMB) (#341088, Millipore-Sigma, Allentown, PA) spectrophotometric assay.^30^ Duplicate samples were digested in 0.5 mg/mL of papain (#P3125, Sigma-Aldrich, St. Louis, MO) at 65°C for 4 hours. Chondroitin-4 sulfate (#C3667, Sigma-Aldrich, St. Louis, MO) was used to establish a standard curve and optical density determined at 525 nm.

### Histology

Cartilage explants (*n* = 1 per treatment) were fixed with 4% paraformaldehyde for 48 hours. After routine histoprocessing for paraffin embedding and sectioning at 4 μm thickness, explants were stained with hematoxylin and eosin (H&E) for tissue architecture analysis and Safranin-O fast green (Saf-O) for examination of proteoglycan. Immunohistochemistry for collagen type II was performed using a monoclonal mouse anti-chick primary antibody (II-II6B3, DSHB, University of Iowa) at a dilution of 1:2000. A blinded, board-certified veterinary pathologist (JBM) scored cartilage explants using a modified-Osteoarthritis Research Society International (OARSI) Scoring system (**Suppl. Table S1**).^31^

Slide scans of Saf O-stained sections were opened in QuPath 0.5.1 and exported in OME TIFF format. In Adobe Photoshop CS6, the images were cropped into individual sections, which were then rotated to a consistent orientation and saved as uncompressed TIFF images. Each section image was opened in MetaMorph 7.10.5.476 software for analysis. A rectangular region of interest (ROI) was created that encompassed approximately two-thirds of the length of sections, which was then centered on each section to mark boundaries along the long axis of the section. A new ROI was created by tracing around the tissue within these boundaries to exclude non-tissue regions from the analysis. A color threshold based on hue, set to exclude hues in the range of 32 to 152 and mark the regions of red Saf-O staining, was applied to the traced ROI. The percent threshold area and average red channel intensity were logged from the “Show Region Statistics” window to an Excel spreadsheet. Line scans were also performed to show the relative levels of red, green, and blue along the width of the section. Percentage of Saf-O stain was generated.

### Gene Expression of Cartilage Explants

Cartilage explants (*n* = 2 per treatment) were snap frozen in liquid nitrogen and biopulverized in liquid nitrogen using a stainless steel biopulverizer (BioSpec Products, Inc, Bartlesville, OK). RNA was extracted using Qiazol lysis reagent^®^ and a commercially available RNA extraction kit (RNeasy Tissue Kit, Qiagen, Germantown, MD). RNA amount and purity was quantified using nanospectrophotometer (NanoDrop™ One, ThermoFisher Scientific, Waltham, MA). Complementary DNA was prepared using a High-Capacity cDNA Reverse Transcription kit (Applied Biosystems, Waltham, MA) and an Eppendorf master cycler. Real-time quantitative PCR was performed using TaqMan™ Master mix (Applied Biosystems, Waltham, MA) and the QuantStudio™ 6 Flex Real-Time PCR System (Applied Biosystems, Waltham, MA). Expression of *IL-6*, *IL-10*, *TNF-α*, *MMP3*, *MMP13*, *ADAMTS4*, *ADAMTS5*, *TGF-β1*, collagen type I (*Coll I*), collagen type II (*Coll II*), proteoglycan 4 (*PRG4*), cartilage oligomeric matrix protein (*COMP*), and aggrecan (*ACAN*) was determined using qRT-PCR and 18S as a reference gene.

### Statistical Analysis

Data was evaluated for normality by assessing histograms for a Gaussian distribution and performing a Shapiro-Wilk test. Parametric quantitative data is presented as mean ± SD. Non-parametric quantitative data is presented as median (range). A Student *t* test or Wilcoxon rank-sum test was used to compare continuous variables between 2 groups. A mixed effects linear model was used to analyze continuous variables with horse as a random effect, and stimulation, treatment and culture media as fixed effects, with the interaction of all 3 probed. A *post hoc* Tukey’s multiple comparison test was used to make pairwise comparisons. Statistical analysis was performed using JMP^®^ Pro 17 (SAS, Cary, NC) software and the level of significance was set at *P* < 0.05.

## Results

### EV Characterization

**Figure 2.**
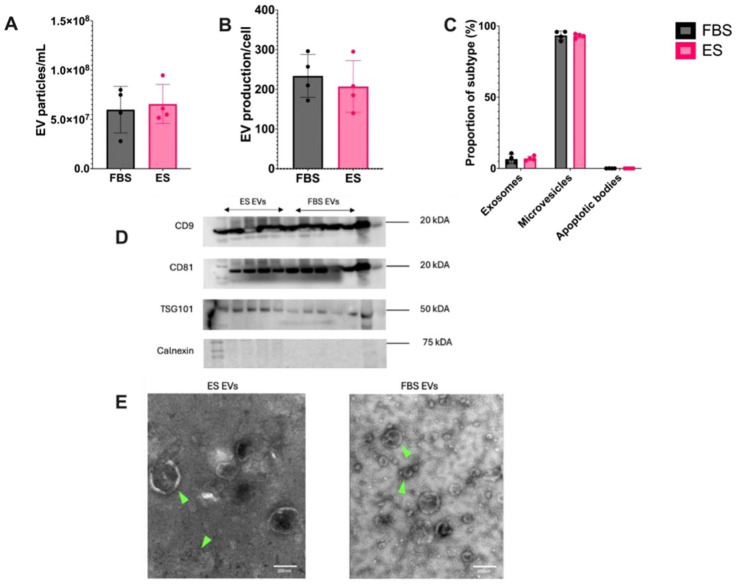
Nanoparticle tracking analysis, Western blot, and transmission electron microscopy characterization of extracellular vesicles (EV) derived from mesenchymal stem cells (MSC) culture expanded in equine serum (ES)-supplemented or fetal bovine serum (FBS)-supplemented media. Nanoparticle tracking analysis EV concentration and subtype data were evaluated using a mixed-effects model with horse as a random effect. (**A**) Mean ± SD number of EV particles produced per sample of stem cells cultured in either ES or FBS-supplemented media. (**B**) Mean ± SD number of EV particles produced per cell cultured in either ES or FBS-supplemented media. (**C**) EV subtype distribution. (**D**) Western blot confirming the presence of positive markers (CD9, CD81, TSG101) and absence of negative marker (Calnexin) on EVs produced from BM-MSCs cultured in ES or FBS-supplemented media. (**E**) Transmission electron microscopy characterization of EVs isolated BM-MSCs cultured in ES or FBS-supplemented media (green arrowheads). Ultrastructural assessment confirmed expected EV size distribution and membrane integrity. Scale bar = 200 nm.

The concentration of EVs and particle size distribution were determined by NTA. No significant differences (*P* = 0.867) were identified in the concentration of EVs isolated from BM-MSCs cultured in FBS or ES (**Fig. 2**). The mean (± SD) particle diameter for EVs from FBS horses and ES horses was 6.62 x 10^7^ nm ± 2.27 x 10^7^ nm and 6.39 x 10^7^ nm ± 1.93 x 10^7^ nm, respectively. There was no difference in the concentration of exosomes, microvesicles or apoptotic bodies in either FBS or ES isolated EVs (**Fig. 2**). Western blot confirmed the presence of specific EV markers including CD9, CD81, and TSG-101. The expression of the transmembrane proteins CD9 and CD81, and the endosomal protein TSG101, in addition to the absence of calnexin, were observed in all samples (**Fig. 2**). Ultrastructural assessment of isolated EVs revealed the expected size distribution and membrane integrity. A heterogeneous population of EVs was detected, corroborating results from the NTA analysis (**Fig. 2**).

### Supernatant Cytokine Analysis

**Figure 3.**
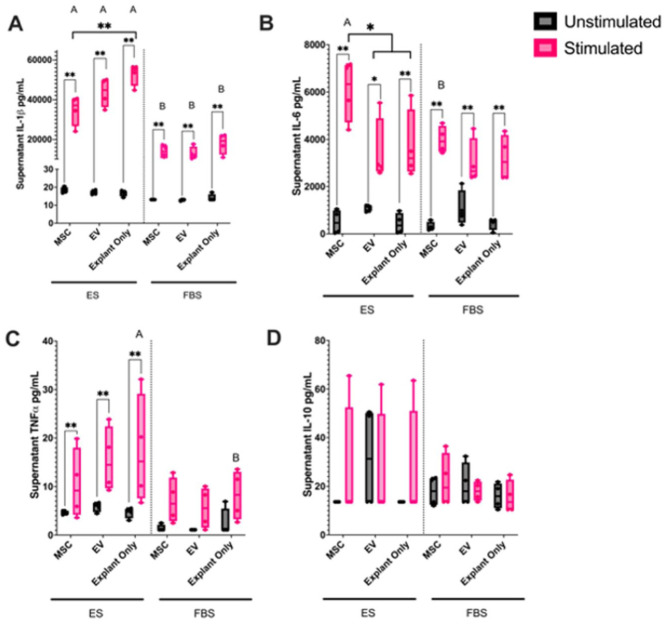
Concentration of (**A**) IL-1β, (**B**) IL-6, (**C**) TNF-α, and (**D**) IL-10 in cell culture supernatants of cartilage explants cultured in ES or FBS-supplemented media with (stimulated) or without (unstimulated) stimulation and treated with MSCs, EVs or left untreated. Differences in supernatant cytokine concentration were evaluated using a mixed-effects model with horse as a random effect. Stimulation, treatment (MSC, EV, explant only), and media (ES or FBS) were considered as fixed effects with their interaction probed in the model. Significant differences between unstimulated and stimulated cultures are shown with connecting bars (*P < 0.05, **P < 0.001); significant differences between treatment groups within a media type are shown with bars between treatment groups (*P < 0.05, **P < 0.001); significant differences between the same treatment groups in different cultures are shown with different letters (*P* < 0.05).

Following culture and treatment, the supernatants were analyzed using a fluorescent bead-based multiplex assay for quantification of IL-1β, IL-6, IL-10, and TNF-α (**Fig. 3**). Stimulation expectedly increased production of IL-1β across all groups (*P* < 0.001). Interestingly, IL-1β supernatant concentrations were significantly lower (*P* < 0.001) in FBS stimulated cultures, compared to ES stimulated cultures regardless of treatment. In ES media, the concentration of IL-1β was significantly decreased (*P* < 0.001) in BM-MSC treated explants compared to explant only cultures.

Stimulation also led to increased production of IL-6 across all groups (*P* < 0.05). Similar to IL-1β, the concentration of IL-6 was significantly lower (*P* < 0.05) in FBS stimulated BM-MSC treated cultures than ES stimulated BM-MSC treated cultures. In ES media, EV treated cultures and explant only cultures had significantly less IL-6 than MSC treated cultures (*P* < 0.05). Furthermore, in FBS media, BM-MSC treated cultures had significantly less IL-6 than BM-MSC treated cultures in ES media (*P* < 0.001).

Stimulation led to a significant increase in TNF-α across all treatment groups in ES media (*P* < 0.001), however, TNF-α was not significantly increased in FBS media cultures. There was also a significant increase in TNF-α in the ES media explant only culture when compared to the explant only culture in FBS media (*P* < 0.05). There was no difference in IL-10 production with stimulation, serum, or treatment.

**Figure 4.**
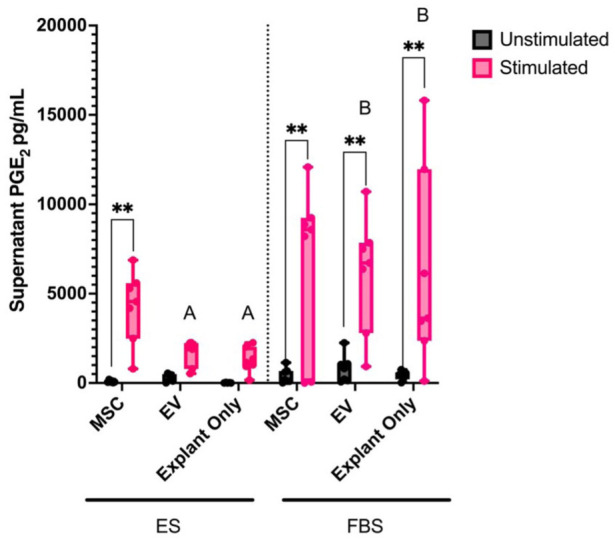
Concentration of PGE2 in cell culture supernatants of cartilage explants cultured in ES or FBS-supplemented media with (stimulated) or without (unstimulated) stimulation and treated with MSCs, EVs or left untreated. Differences in supernatant cytokine concentration were evaluated using a mixed-effects model with horse as a random effect. Stimulation, treatment (MSC, EV, explant only), and media (ES or FBS) were considered as fixed effects with their interaction probed in the model. Significant differences between unstimulated and stimulated cultures are shown with connecting bars (*P < 0.05, ** *P* < 0.001); significant differences between treatment groups within a media type are shown with bars between treatment groups (*P < 0.05, **P < 0.001); significant differences between the same treatment groups in different cultures are shown with different letters (*P* < 0.05).

Stimulation led to a significant increase in supernatant PGE2 concentrations in all treatment groups cultured in FBS media, while in ES media only BM-MSC treated explant cultures had increased PGE2 following stimulation (*P* < 0.001). In addition, PGE2 concentrations were significantly decreased in ES media EV treated explants and explant only cultures to the corresponding groups in FBS media (*P* < 0.001) (**Fig. 4**).

### Glycosaminoglycan Quantification

**Figure 5.**
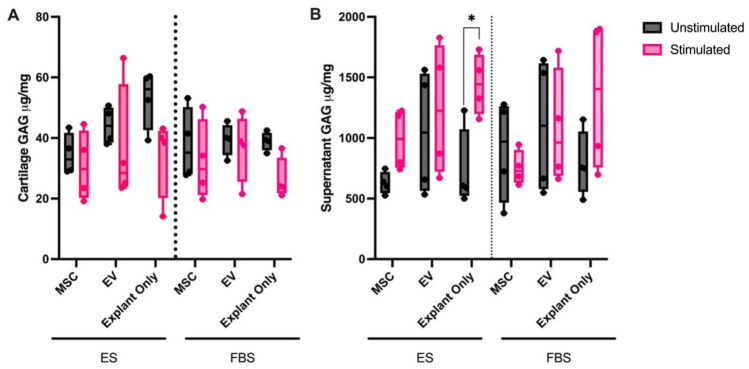
Concentration of glycosaminoglycan (GAG) in (**A**) cell culture supernatants and (**B**) cartilage explants that were cultured in ES or FBS-supplemented media with (stimulated) or without (unstimulated) stimulation and treated with MSCs, EVs or left untreated. Differences in supernatant cytokine concentration were evaluated using a mixed-effects model with horse as a random effect. Stimulation, treatment (MSC, EV, explant only), and media (ES or FBS) were considered as fixed effects with their interaction probed in the model. Significant differences between unstimulated and stimulated cultures are shown with connecting bars (*P < 0.05).

Glycosaminoglycan was quantified in both culture supernatants and papain-digested cartilage explants to investigate degradation of the ECM. There was significantly more GAG in the supernatants of stimulated explant only cultures in ES media compared to unstimulated cultures (*P* < 0.05) (**Fig. 5**). No other significant differences were noted in supernatant GAG concentrations. There were no significant differences in the amount of GAG in cartilage explants regardless of stimulation, serum, or treatment (**Fig. 5**).

### Histology

**Figure 6.**
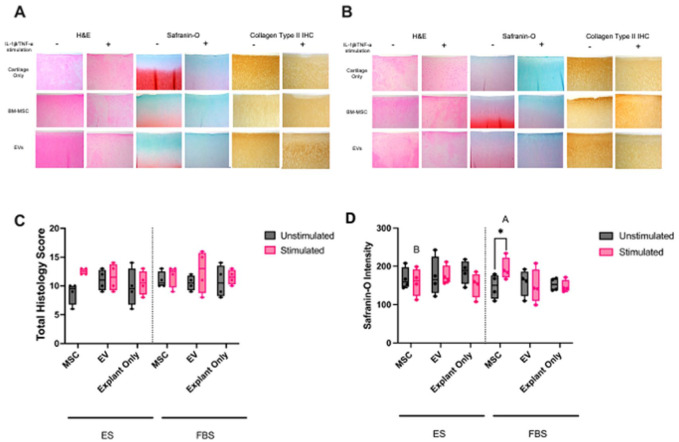
Photomicrographs of cartilage explants in (**A**) ES-supplemented media and (**B**) FBS-supplemented media showing tissue architecture (H&E staining), proteoglycan content (Safranin-O staining), and collagen type II presence (collagen type II IHC) with and without stimulation (scale bar = 100 µm). (**C**) Histopathology scores of cartilage explants. (**D**) Intensity of Safranin-O staining in cartilage explants. Stimulation, treatment (MSC, EV, explant only), and media (ES or FBS) were considered as fixed effects with their interaction probed in the model. Significant differences between unstimulated and stimulated cultures are shown with connecting bars (*P < 0.05); significant differences between the same treatment groups in different cultures are shown with different letters (*P* < 0.05).

Representative histologic images and semi-quantitative total histopathology scoring is shown in **Figure 6**. Histopathology scores by individual categories are shown in **Suppl. Table S2**. While total histopathology scores were increased by stimulation in all treatment groups, these increases were not significant. There was also no significant effect of serum or treatment. Quantification of Saf-O staining intensity revealed significantly more Saf-O staining intensity in stimulated BM-MSC treated explant cultures in FBS compared to ES (**Fig. 6**). No other significant effects of serum or treatment were noted.

### Gene Expression

**Figure 7.**
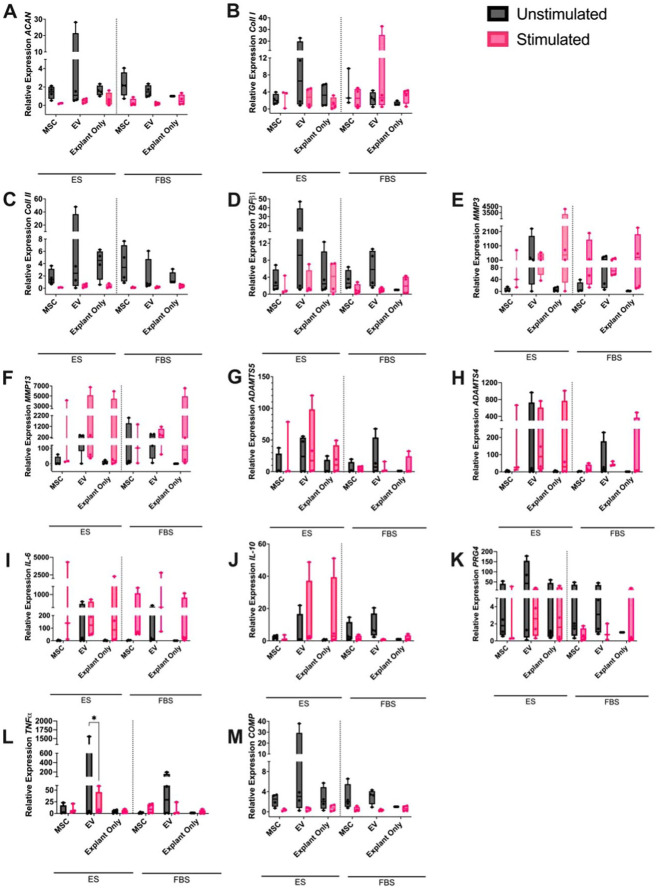
Relative expression of (**A**) *ACAN*, (**B**) *Coll I*, (**C**) *Coll II*, (**D**) *TGF-b1*, (**E**) *MMP3*, (**F**) *MMP13*, (**G**) *ADAMTS5*, (**H**) *ADAMTS4*, (**I**) *IL-6*, (**J**) *IL-10*, (**K**) *PRG4*, (**L**) *TNF-α*, and (**M**) *COMP* in unstimulated and stimulated cartilage explants treated with BM-MSCs, EVs or left untreated cultured in either ES or FBS-supplemented media. Differences in supernatant cytokine concentration were evaluated using a mixed-effects model with horse as a random effect. Stimulation, treatment (MSC, EV, explant only), and media (ES or FBS) were considered as fixed effects with their interaction probed in the model. Significant differences between unstimulated and stimulated cultures are shown with connecting bars (*P < 0.05).

qRT-PCR analysis of the cartilage explants demonstrated no significant effect of stimulation, serum, or treatment on the expression of *IL-6*, *IL-10*, *MMP3*, *MMP13*, *ADAMTS4*, *ADAMTS5*, *TGF-β1*, *Coll I*, *Coll II*, *PRG4*, *COMP*, or *ACAN*. There was significantly more *TNF-α* expression in unstimulated EV treated cells in ES media compared to all other conditions (*P* < 0.05) (**Fig. 7**).

## Discussion

In recent years, there has been an increasing emphasis on the reduction of inflammation and using immunomodulation as key strategies in the management of OA. Studies have demonstrated the immunomodulatory effects of MSCs and have linked these to the MSC secretome.^13,32^ Extracellular vesicles make up a large portion of the secretome, containing key biocargo that modulates responses of surrounding cells; therefore, we sought to compare the protective effects of MSCs to EVs in an *in vitro* model of OA. In addition, we examined the effects of serum type on the culture system. In this study, although serum type did not affect EV characterization parameters, serum type did have some impacts on cartilage inflammation and we found that BM-MSCs suppressed inflammation to a greater degree than EVs.

Quantification of immunomodulatory mediators in culture supernatants showed that co-culture of cartilage explants with BM-MSCs in ES significantly reduced IL-1β compared to the cartilage explant control, while EVs did not. Co-cultures of cartilage explants with BM-MSCs in ES also had significantly more IL-6 than both EV co-cultures and cartilage explant controls. These findings suggest that BM-MSCs, but not EVs, modulate the response of cartilage to inflammatory stimuli when cultured in equine serum. The observed alterations in the cytokine profile of the supernatants could reflect cytokine release from BM-MSCs, cartilage explants, or both. While further work would be needed to decipher the source of cytokines produced in a co-culture system, we believe that the overall media profile is critical when examining immunomodulation that would occur in a joint. Although IL-1β is well recognized for its pro-inflammatory role in the joint, IL-6 may serve a more nuanced, immunomodulatory function. IL-6 is a pleiotropic gene with both pro- and anti-inflammatory effects depending on whether classic signaling or trans-signaling is occurring.^33^ IL-6 appears to have pro-chondrogenic and anabolic roles in equine and human joint tissues.^34,35^ In addition, IL-6 production by BM-MSCs is associated with macrophage polarization toward an anti-inflammatory phenotype (M2).^34^

The lack of immunomodulation by EVs in this study is intriguing. It is possible that the EVs used did not contain the “ideal” biocargo to dampen inflammation in our culture system. Other studies have demonstrated that pre-conditioning or priming MSCs with inflammatory mediators such as IL-1β, TNF-α, or IFN-g can enhance their immunomodulatory capabilities^36^ and can also increase the immunomodulatory capabilities of the EVs they produce.^37,38^ Other studies have demonstrated that the cell source of EVs affects their immunomodulatory properties.^38,39^ For example, EVs derived from human MSCs were found to decrease the anabolic function of chondrocytes, however, this effect was rescued when EVs were derived from MSCs engineered to express miR-140-5p.^38^ Zhu *et al*.^39^ also found that EVs from induced pluripotent stem cells (iPS) were more chondroprotective than EVs derived from synovial MSCs in a mouse model of collagenase-induced OA. Another factor to consider is the distribution of EV subtype in this study as our NTA showed that the majority of EVs isolated from BM-MSCs were microvesicles (100-1,000 nm). Since studies have demonstrated a significant difference in the characteristics and biocargo of exosomes and microvesicles, including membrane lipid, RNA and proteins,^40,41^ further investigation into the bioactivity of EV subtype is of interest. Finally, while the dose of EVs required to achieve a therapeutic effect is unknown, previous studies have demonstrated increasing chondroprotective effects with increasing dose.^42^ It is possible that the dose used in this study may have been too low to elicit downstream effects. Further studies investigating the optimal source and dose of EVs are necessary.

Serum type affected the response of cartilage explants to inflammatory stimuli. Previous studies have also demonstrated effects of serum supplementation on the proliferation, differentiation and chondroprotective properties of MSCs.^43^ Overall, supernatants from ES cultures contained higher concentrations of IL-1β, IL-6, and TNF-α when compared to FBS cultures. Supernatant concentrations of IL-1β were significantly higher in all ES stimulated cultures compared to FBS stimulated cultures, while BM-MSC co-cultures had significantly higher IL-6 in ES media compared to FBS media, and cartilage explants alone had significantly higher TNF-α in ES media compared to FBS media. Similar results were found in a previous study in which BM-MSCs cultured in ES had significantly higher concentrations of IL-6 upon stimulation compared to cells cultured in FBS and platelet lysate.^27^ Once again, it is interesting to consider the mixed downstream effects of IL-6 because in this previous study, we also found that BM-MSCs cultured in ES were the most effective at suppressing T cell proliferation compared to BM-MSCs cultured in FBS and platelet lysate. Notably, although the supernatant concentrations of several cytokines were higher in ES cultures, we found that PGE2 was higher in FBS cultures. PGE2 is an important pro-inflammatory mediator in osteoarthritis^44^ and has been shown to be associated with the pain response in inflamed joints.^45^ However, MSC production of PGE2 is also an important pathway in suppression of T cell activity and it is likely that low concentrations of PGE2 would be of benefit both *in vitro* and *in vivo*.^46^ A previous study found that culture of MSCs in serum-free media decreased production of PGE2 by MSCs when compared to FBS media confirming that media type does alter MSC properties and secretion of inflammatory mediators.^24^ However, in the current study, because all treatment groups in FBS media had higher levels of PGE2, not just BM-MSC co-cultures, the majority of PGE2 appears to be produced by chondrocytes, or contained within the FBS itself.

While changes in the culture supernatants detected in this study support immunomodulation by BM-MSCs, no significant changes were noted in chondrocyte gene expression, GAG content or cartilage histology across treatment groups. While stimulation of cartilage explants did tend to increase expression of catabolic genes and decrease expression of anabolic genes, increase loss of GAG, and increase histopathology scores these findings were not statistically significant. None of these parameters were affected by treatment with BM-MSCs or EVs regardless of serum type. It is possible that a longer culture period or different dose of BM-MSCs or EVs is necessary to appreciate changes in the cartilage explants themselves, not just the supernatant. In addition, there was notable inter-animal variability in gene expression and supernatant cytokines, therefore, we may not have been able to detect differences due to a small sample size. Furthermore, subtle (grossly inapparent) microscopic differences among cartilage explant sites at the time of harvest may have also introduced inherent variability impacting molecular and morphologic parameters regardless of treatment or culture media. Hence, any inter-horse or inter-site variability impacting the architectural integrity of the explants or the quality of both BM-MSCs and EVs remains a significant limitation of this study, an inherent limitation referenced in other donor-based equine studies investigating MSCs and EVs.^47,48^ Moreover, the *in vitro* nature of the study, focusing solely on cartilage without the inclusion of synoviocytes, subchondral bone, and synovial fluid, does not fully represent the complexities of intra-articular interactions *in vivo*. Finally, only a select group of cytokines and genes were evaluated. It is well known that osteoarthritis is driven by complex signaling pathways, and therefore the full effects of BM-MSCs and EVs may not have been captured in the assays included in this study.

## Conclusion

This study provides evidence suggesting that equine BM-MSCs appear to have enhanced immunomodulatory properties compared to EVs derived from BM-MSCs when used to treat stimulated cartilage explants. However, this effect was only found in cultures supplemented with ES, not FBS. Overall, cultures supplemented with FBS had lower concentrations of immunomodulatory cytokines but higher concentrations of PGE2. While some beneficial alterations in culture supernatants were detected, we were not able to detect any significant protective effects of either BM-MSCs or EVs on the cartilage explants. Finally, we did not find any significant effect of serum type on EVs produced from cultured BM-MSCs.

Further investigation of the effect of both BM-MSCs and EVs and the effect of serum type is warranted including examination of EV source and dosage and the effect of MSC priming on EV functionality.

## Supplemental Material

sj-docx-1-car-10.1177_19476035251378693 – Supplemental material for Equine Bone Marrow–Derived MSCs and Their EVs Exhibit Different Immunomodulatory Effects on Cartilage Explants in an In Vitro Osteoarthritis Model Supplemental material, sj-docx-1-car-10.1177_19476035251378693 for Equine Bone Marrow–Derived MSCs and Their EVs Exhibit Different Immunomodulatory Effects on Cartilage Explants in an In Vitro Osteoarthritis Model by Angela M. Gaesser, Justine M. Cianci, Kayla Even, Renata L. Linardi, Gordon Ruthel, Dhvani Barot, Hoda Elkhenany, Julie B. Engiles and Kyla F. Ortved in CARTILAGE

sj-docx-2-car-10.1177_19476035251378693 – Supplemental material for Equine Bone Marrow–Derived MSCs and Their EVs Exhibit Different Immunomodulatory Effects on Cartilage Explants in an In Vitro Osteoarthritis Model Supplemental material, sj-docx-2-car-10.1177_19476035251378693 for Equine Bone Marrow–Derived MSCs and Their EVs Exhibit Different Immunomodulatory Effects on Cartilage Explants in an In Vitro Osteoarthritis Model by Angela M. Gaesser, Justine M. Cianci, Kayla Even, Renata L. Linardi, Gordon Ruthel, Dhvani Barot, Hoda Elkhenany, Julie B. Engiles and Kyla F. Ortved in CARTILAGE

sj-docx-3-car-10.1177_19476035251378693 – Supplemental material for Equine Bone Marrow–Derived MSCs and Their EVs Exhibit Different Immunomodulatory Effects on Cartilage Explants in an In Vitro Osteoarthritis Model Supplemental material, sj-docx-3-car-10.1177_19476035251378693 for Equine Bone Marrow–Derived MSCs and Their EVs Exhibit Different Immunomodulatory Effects on Cartilage Explants in an In Vitro Osteoarthritis Model by Angela M. Gaesser, Justine M. Cianci, Kayla Even, Renata L. Linardi, Gordon Ruthel, Dhvani Barot, Hoda Elkhenany, Julie B. Engiles and Kyla F. Ortved in CARTILAGE
