# Supplementary material for: Equine Bone Marrow–Derived MSCs and Their EVs Exhibit Different Immunomodulatory Effects on Cartilage Explants in an In Vitro Osteoarthritis Model
Source: Cartilage. 2025 Sep 25:19476035251378693. Online ahead of print. doi: 10.1177/19476035251378693 (PMC12463865; doi:10.1177/19476035251378693)

**Supplementary Figure 1:** Full-length western blot confirming the presence of positive markers a) CD9, b) CD81, c) TSG101 and absence of negative marker d) Calnexin on EVs produced from BM-MSCs cultured in ES or FBS-supplemented media. The cropped area represented in Figure 2 is marked on each gel with a red rectangle.


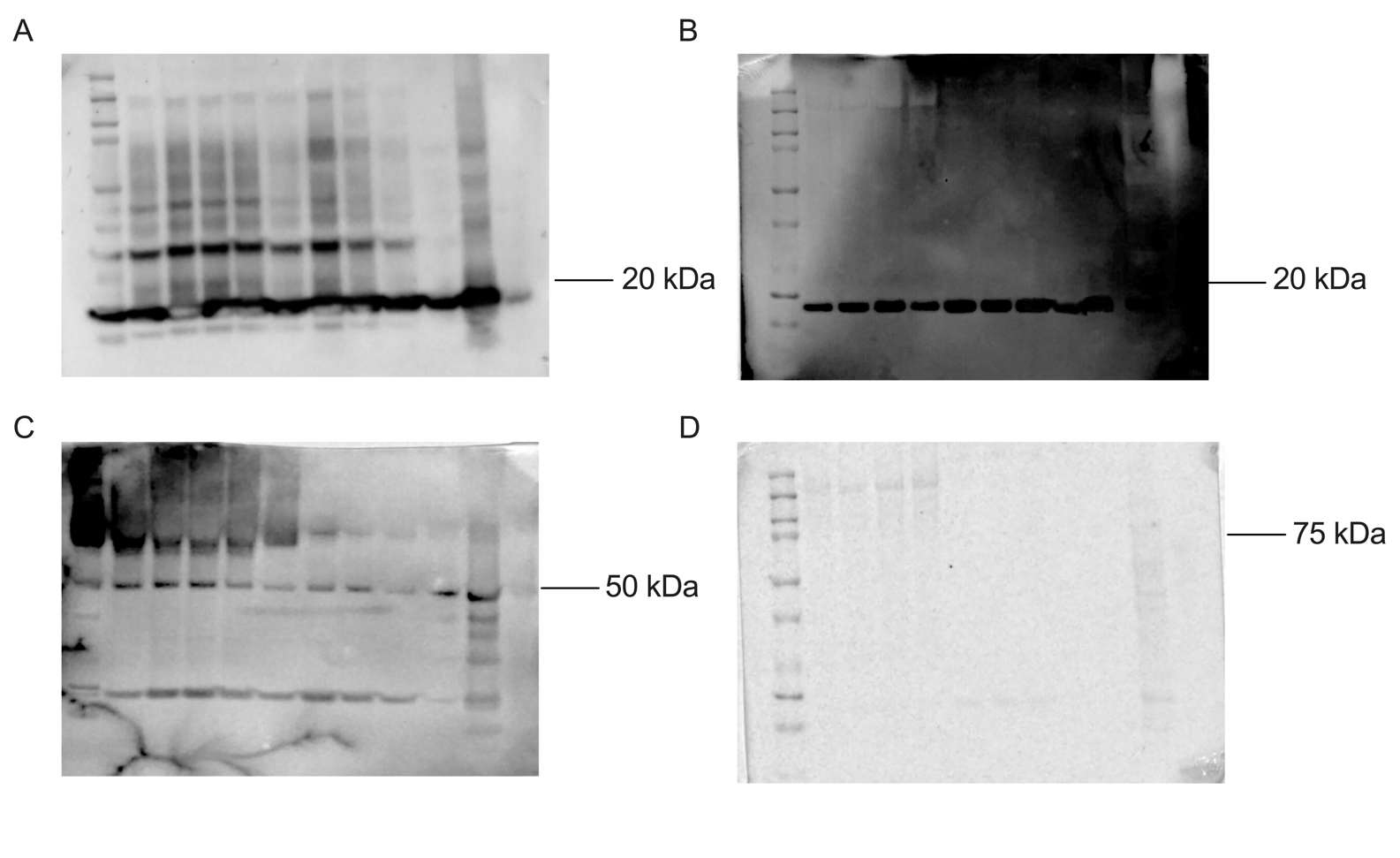

Supplement: sj-docx-1-car-10.1177_19476035251378693 – Supplemental material for Equine Bone Marrow–Derived MSCs and Their EVs Exhibit Different Immunomodulatory Effects on Cartilage Explants in an In Vitro Osteoarthritis Model [file sj-docx-1-car-10.1177_19476035251378693.docx]
