# Supplementary material for: Equine Bone Marrow–Derived MSCs and Their EVs Exhibit Different Immunomodulatory Effects on Cartilage Explants in an In Vitro Osteoarthritis Model
Source: Cartilage. 2025 Sep 25:19476035251378693. Online ahead of print. doi: 10.1177/19476035251378693 (PMC12463865; doi:10.1177/19476035251378693)
Supplement: sj-docx-2-car-10.1177_19476035251378693 – Supplemental material for Equine Bone Marrow–Derived MSCs and Their EVs Exhibit Different Immunomodulatory Effects on Cartilage Explants in an In Vitro Osteoarthritis Model [file sj-docx-2-car-10.1177_19476035251378693.docx]

**Supplementary Table 1.** Scoring system for histological tissue pathology adapted from McIlwraith et al. (2010).^1^

| **Outcome Parameter** | **Score** | **Description** |
| --- | --- | --- |
| **Chondrocyte necrosis*** | 0 | Normal section without necrosis within 150μm of articular surface |
|  | 1 | 1-5 necrotic cells located within 150μm of articular surface per 20x objective |
|  | 2 | 6-9 necrotic cells located within 150μm of articular surface per 20x objective |
|  | 3 | 10-15 necrotic cells located within 150μm of articular surface per 20x objective |
|  | 4 | >15 necrotic cells located within 150μm of articular surface per 20x objective |
| **Cluster (complex chondrone) formation** | 0 | No cluster formation within 150μm of articular surface |
|  | 1 | Two chondrocytes (doublets) within same lacunae within 150μm of articular surface |
|  | 2 | 2-3 chondrocytes (doublets & triplets) within 150μm of articular surface |
|  | 3 | 3-4 chondrocytes within same lacunae within 150μm of articular surface |
|  | 4 | > 4 chondrocytes within same lacunae within 150μm of articular surface |
| **Fibrillation/fissuring** | 0 | No fibrillation/fissuring of the articular cartilage surface |
|  | 1 | Fibrillation/fissuring of the articular cartilage surface restricted to surface and superficial zone |
|  | 2 | Fissuring that extends into the middle zone |
|  | 3 | Fissuring that extends to the level of the deep zone |
|  | 4 | Fissuring that extends into the deep zone |
| **Focal cell loss*** | 0 | Normal cell population within 150μm of articular surface |
|  | 1 | A 10-20% area of acellularity within 150μm of articular surface per 20x field |
|  | 2 | A 20-30% area of acellularity within 150μm of articular surface per 20x field |
|  | 3 | A 40-50% area of acellularity within 150μm of articular surface per 20x field |
|  | 4 | A >50% area of acellularity within 150μm of articular surface per 20x field |
| **Safranin-O stain uptake** | 0 | Normal staining |
|  | 1 | <25% loss of staining characteristics |
|  | 2 | 25-50% loss of staining characteristics |
|  | 3 | 50-75% loss of staining characteristics |
|  | 4 | Greater than 75% loss of staining characteristics |
| **Collagen type II IHC** | 0 | Normal staining |
|  | 1 | <25% loss of staining characteristics |
|  | 2 | 25-50% loss of staining characteristics |
|  | 3 | 50-75% loss of staining characteristics |
|  | 4 | Greater than 75% loss of staining characteristics |

* Chondrocyte necrosis is used to grade presence of lacunae with necrotic nuclei still present compared to focal cell loss which is most likely an extension of the pathologic change, but lacunae or nuclei are no longer present.
