# Supplementary material for: Equine Bone Marrow–Derived MSCs and Their EVs Exhibit Different Immunomodulatory Effects on Cartilage Explants in an In Vitro Osteoarthritis Model
Source: Cartilage. 2025 Sep 25:19476035251378693. Online ahead of print. doi: 10.1177/19476035251378693 (PMC12463865; doi:10.1177/19476035251378693)
Supplement: sj-docx-3-car-10.1177_19476035251378693 – Supplemental material for Equine Bone Marrow–Derived MSCs and Their EVs Exhibit Different Immunomodulatory Effects on Cartilage Explants in an In Vitro Osteoarthritis Model [file sj-docx-3-car-10.1177_19476035251378693.docx]

**Supplementary Table 2.** Histologic scoring of cartilage explants following culture in different conditions with and without IL-1β/TNFα stimulation.

| **Media** | **Treatment group** | **Stimulation** | **Chondrocyte necrosis** | **Chondrone formation** | **Fibrillation / fissuring** | **Focal cell loss** | **Safranin-0 staining %** | **Collagen type II IHC % staining** | **Total score** |
| --- | --- | --- | --- | --- | --- | --- | --- | --- | --- |
| FBS | Cartilage only | Unstimulated | 3 ± 0.71 | 1 ± 0.58 | 0.25 ± 0.25 | 1.5 ± 0.29 | 3.25 ± 0.48 | 1.75 ± 0.25 | 10.75 ± 1.38 |
|  |  | Stimulated | 3 ± 0.41 | 1 ± 0.00 | 0.25 ± 0.25 | 2 ± 0.41 | 3.75 ± 0.25 | 1.5 ± 0.29 | 11.5 ± 0.65 |
|  | BM-MSC | Unstimulated | 3.25 ± 0.25 | 0.5 ± 0.29 | 0.25 ± 0.25 | 1.75 ± 0.25 | 3.25 ± 0.48 | 1.5 ± 0.65 | 10.5 ± 0.65 |
|  |  | Stimulated | 3.25 ± 0.48 | 1.75 ± 0.48 | 0.5 ± 0.29 | 2.75 ± 0.25 | 2.75 ± 0.63 | 1.75 ± 0.25 | 12.5 ± 1.85 |
|  | EV | Unstimulated | 3.5 ± 0.29 | 0.5 ± 0.29 | 0.25 ± 0.25 | 1.75 ± 0.48 | 3.25 ± 0.48 | 1.75 ± 0.25 | 11 ± 0.70 |
|  |  | Stimulated | 3 ± 0.71 | 0.75 ± 0.25 | 0.5 ± 0.29 | 2.25 ± 0.63 | 3.5 ± 0.5 | 1.5 ± 0.29 | 11.75 ± 0.95 |
| ES | Cartilage only | Unstimulated | 2.5 ± 0.5 | 1 ± 0.41 | 0.75 ± 0.25 | 2 ± 0.41 | 2.5 ± 0.5 | 1 ± 0 | 9.75 ± 1.65 |
|  |  | Stimulated | 3.75 ± 0.25 | 0.25 ± 0.25 | 0.25 ± 0.25 | 2 ± 0 | 2.75 ± 0.63 | 1.5 ± 0.29 | 10.5 ± 1.04 |
|  | BM-MSC | Unstimulated | 3 ± 0.71 | 1 ± 0.58 | 0.25 ± 0.25 | 1.75 ± 0.25 | 3.5 ± 0.29 | 1.5 ± 0.5 | 11 ± 0.91 |
|  |  | Stimulated | 3.5 ± 0.29 | 0.5 ± 0.29 | 0 ± 0 | 2.75 ± 0.63 | 3 ± 0.41 | 1.75 ± 0.25 | 11.5 ± 1.19 |
|  | EV | Unstimulated | 2 ± 0.58 | 1 ± 0.41 | 0.25 ± 0.25 | 1.25 ± 0.25 | 3 ± 0.41 | 1.25 ± 0.25 | 8.75 ± 0.95 |
|  |  | Stimulated | 3.5 ± 0.29 | 1.25 ± 0.63 | 0 ± 0 | 2.75 ± 0.25 | 3.25 ± 0.48 | 1.75 ± 0.25 | 12.5 ± 0.29 |

IHC, immunohistochemistry.

Data presented as mean ± SEM. *n* = 4 horses with 2 experimental replicates per horse.
